# Identification and functional characterization of a novel nonsense mutation of *CASR* gene in a familial hypocalciuric hypercalcemia pedigree

**DOI:** 10.1016/j.gendis.2025.101781

**Published:** 2025-07-25

**Authors:** Yang Tian, Bingyang Liu, Yang Xudan, Ruojun Qiu, Zhang Shaojun, Huang Haoshu, Wu Fang, Fenping Zheng

**Affiliations:** a Department of Endocrinology, The Fourth Affiliated Hospital, Zhejiang University School of Medicine, N1 ShangCheng Road, Yiwu, Zhejiang 322000, China; b Department of Endocrinology, The Affiliated Sir Run Run Shaw Hospital, School of Medicine, Zhejiang University, 3 East QingChun Rd, Hangzhou, Zhejiang 310016, China; c Department of Respiratory and Critical Care Medicine, The Fourth Affiliated Hospital, School of Medicine, Zhejiang University, N1 ShangCheng Road, Yiwu, Zhejiang 322000, China

Familial hypocalciuric hypercalcemia (FHH), a rare cause of hypercalcemia, features a benign, lifelong mild-to-moderate hypercalcemia. It is often misdiagnosed as primary hyperparathyroidism, so patients may wrongly undergo parathyroidectomy. Thus, it is important to raise the suspicion of FHH before the diagnosis of primary hyperparathyroidism. Familial hypocalciuric hypercalcemia 1 (FHH1) is caused by heterozygous inactivating mutations of calcium-sensing receptor (CaSR) gene, and CaSR is a 1078-amino acid G-protein-coupled receptor and is mostly expressed in the parathyroid gland and renal tubule to maintain the homeostasis of calcium by regulating parathyroid hormone secretion and urinary calcium excretion. So far, over 150 different germline mutations of *CASR* in FHH1 have been reported, with 85% being missense mutations and only 3%–4% being nonsense mutations.^1^^,^^2^ In this study, we report a novel heterozygous nonsense mutation on the *CASR* (c.1799 G > A, p.Trp600∗) gene; this variant is predicted to cause an amino acid alteration from tryptophan to a premature stop codon in the extra-cellular domain, and the affected proband presented with mild hypercalcemia, low calcium to creatinine clearance ratio, and hyperglycemia with reserved insulin secreting function.

We firstly conducted pedigree analysis including clinical manifestations, laboratory tests characteristics (Fig. 1A; Table S1), and mutation site of *CASR* gene verification (Fig. 1B), and our results confirmed this nonsense mutation in the *CASR* gene segregated with the clinical traits of FHH1; we also found two siblings of the proband, who harbored *CASR* mutation, were also afflicted with hyperglycemia with reserved insulin secreting function (Table S2).

**Figure 1:**
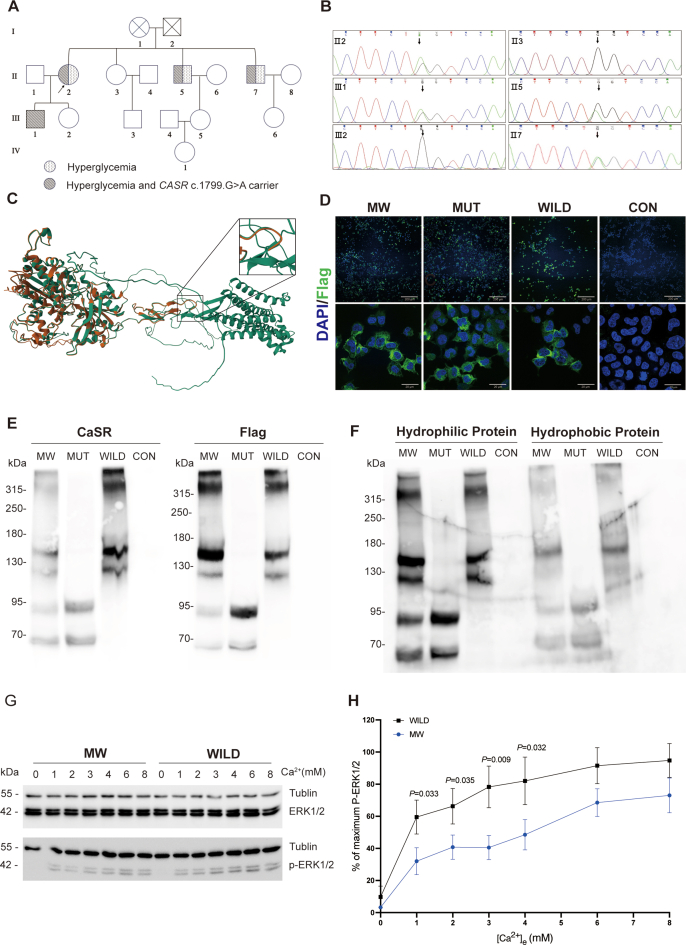
A novel nonsense mutation of *CASR* gene is associated with FHH1. **(A)** Pedigree of a family with FHH1. The squares represent male and the circles represent female family members. Diabetes mellitus-affected individuals are represented by speckled symbols, and hypercalcemia together with *CASR* mutant are represented by slash shading symbols. The index case is marked by an arrow. **(B)** Sanger sequencing results of exon 7 of the *CASR* gene in the family members. Subjects Ⅱ2, Ⅱ5, Ⅱ7, and Ⅲ1 carried the heterozygous *CASR* mutation and concurrently exhibited hypercalcemia, which demonstrates that the phenotype segregates with the genotype within this pedigree. **(C)** Effect of *CASR* mutation on the modeled structure of the calcium sensing receptor. The red and green ribbons represent mutant and wild monomeric CaSR, respectively, compared with the wild-type c.1799 G > A, which caused a truncated protein in the extracellular domain of the CaSR receptor. **(D)** Cellular expression of Flag in transfected HEK293T cells. Confocal microscopy images showed that Flag (green) was localized to the periphery of MW, MUT, and WILD, but not in CON. Nuclei were labeled with DAPI (blue). Scale bar: 10 μm. The Flag signals were confirmed at the membrane periphery. **(E)** Western blotting analysis of total protein. In the MW and MUT groups, two distinct bands were detected below 70 KDa and 95 KDa, corresponding to Flag and CaSR proteins, respectively. In contrast, these bands were not observed in the WILD and CON groups. **(F)** Western blotting analysis of hydrophilic and hydrophobic proteins. In the western blotting profiles of hydrophobic proteins from the MW and MUT groups, two relatively faint bands were identified below 70 KDa and 95 KDa. This observation indicates the expression of truncated protein forms on the cell membrane surface. **(G)** MAPK activity response to eCa^2+^. MW showed a blunted response to eCa^2+^ compared with WILD. **(H)** Effect of different eCa^2+^ concentrations on phosphorylation of ERK1/2 of MW and WILD. Concentrations of eCa^2+^ (0, 1, 2, 3, 4, 6, 8 mM) were plotted on the *x*-axis, the ratio of pERK1/2 over total ERK1/2, expressed as a percentage of the maximum response value for each concentration measured based on band intensity, was on the *y*-axis. Subsequently, the two curves were compared (values were plotted as mean ± standard error of the mean; *p*-values indicate the unpaired MW *t*-test on WILD for each concentration). The data presented were the cumulative results of four independent biological replicates. CASR, calcium sensing receptor; FHH1, familial hypocalciuric hypercalcemia 1; MW, HEK293 cells transfected with 1 μg mutant *CASR* plasmid together with 1 μg wild *CASR* plasmid; MUT, HEK293 cells transfected with 2 μg mutant *CASR* plasmid; WILD, HEK293 cells transfected with 2 μg wild *CASR* plasmid; CON, HEK293 cells transfected with 2 μg of control plasmid; MAPK, mitogen-activated protein kinase; ERK1/2, extracellular signal-regulated kinase 1/2.

According to the American College of Medical Genetics and Genomics (ACMG) guideline, this nonsense mutation was predicted to be pathogenic. An online SWISS-MODEL workspace (https://swissmodel.expasy.org) was employed to predict the effect of this variant on the protein structure of CaSR, demonstrating that this alteration will lead to a truncated CaSR protein in the extracellular domain (Fig. 1C), with a predicted molecular weight of 66.04 kDa.

Because a large proportion of nonsense mutations are prone to undergo nonsense-mediated decay, we performed an *in vitro* study of this mutation to assess membrane expression. We constructed a 3flag-tagged human *CASR* W600∗ plasmid through overlap PCR and transfected HEK293 cells with 1 μg mutant *CASR* plasmid together with 1 μg wild *CASR* plasmid (MW) to mimic heterozygous mutation of *CASR*, 2 μg mutant *CASR* plasmid (MUT), 2 μg wild *CASR* plasmid (WILD), and 2 μg of control plasmid (CON). Western blotting of total protein extracted from the four groups using anti-CaSR (ThermoFisher: MA1-934, corresponding to amino acids 215–235 of human CaSR) and anti-Flag antibody showed immunoreactive bands lower than 70 kDa and 95 kDa in MW and MUT corresponding to the truncated monomeric form of CaSR (Fig. 1E). Hydrophobic and hydrophilic proteins extracted and enriched by CelLytic™ MEM Protein Extraction Kit (Sigma–Aldrich: CE0050) were also subjected to western blotting, and faint immunoreactive bands lower than 70 kDa and 95 kDa in hydrophobic protein indicate the membrane expression of this truncated CaSR (Fig. 1F). Fluorescence immunocytochemistry assay in MW, MUT, and WILD displayed similarly specific staining using anti-Flag antibody (green), while cells in CON showed no green fluorescence signal, confirming cell membrane expression of flag-tagged CaSR (Fig. 1D). We then accessed mitogen-activated protein kinase (MAPK) responsiveness to investigate transduction activity of CaSR,^3^ in both MW and WILD group, and observed evident phosphorylation of extracellular signal-regulated kinase 1/2 (ERK1/2) when the eCa^2+^ was elevated from 0 to 8 mM but a blunted phosphorylation response of ERK1/2 of the MW group compared with the WILD group (Fig. 1G and H). This indicated that the heterozygous mutation of *CASR* W600∗ resulted in hypoactivation of the receptor and therefore failed to maintain the homeostasis of calcium.

We also reviewed *CASR* mutations in FHH1. So far, 14 distinct nonsense mutations, including our case, have been reported. Among them, 8 out of 14 mutations are located in exon 7, with an average S–Ca of 2.827 ± 0.047 mM (presented as mean ± standard error of the mean). The other 6 mutations occur in exons 2–4, are predicted to undergo nonsense-mediated decay, and show milder hypercalcemia (average S–Ca 2.625 ± 0.024 mM) (Table S3). The CaSR exists as homodimers on the cell surface. Its extracellular domain forms a Venus flytrap structure and contains 5 calcium-binding sites (CaBS-1 to 5), and its dimerization is essential for normal CaSR function. In nonsense mutations, premature stop codons located 50–55 bp upstream of the final exon–exon junction will be subjected to nonsense-mediated decay, causing haploinsufficiency and a milder phenotype. In contrast, premature stop codons in the gene's 3′ region, including the last exon and ∼55 bp of the penultimate, evade nonsense-mediated decay, yielding truncated proteins. These truncated proteins disrupt normal CaSR dimerization, exerting a dominant-negative effect. This mechanism explains the variable severity of hypercalcemia caused by *CASR* nonsense mutations at different genomic locations. This might partly explain the lower incidence of nonsense mutations in FHH1 compared with autosomal dominant hyperparathyroidism, as the mild phenotype caused by a nonsense mutation subjected to nonsense-mediated decay could be overlooked.

Although FHH1 has long been regarded as a benign and non-threatening condition, an in-depth data analysis by Ridge Dershem^4^ reveals that *CASR* variants are associated with a range of health issues including cardiovascular problems such as embolism, thrombosis, and chronic diastolic heart failure, as well as neurological disorders including dementia, major depression, pancreatitis, and a propensity for alcohol abuse, underlining the need for a more comprehensive understanding and vigilant monitoring of FHH1 and its related gene variants. Interestingly, within this family, our findings revealed that, except for the younger patient III:1, the *CASR* W600∗ harboring individuals also exhibited type 2 diabetes mellitus with reserved insulin secretion. This is notable because relatively few prior studies have documented the association between FHH1 and hyperglycemia. Insulin is the major hypoglycemic hormone. Previous investigations on small cohorts of individuals with FHH1 have documented that their glucose tolerance test outcomes and insulin release test profiles exhibit no significant difference from the general population, and glucagon-like peptide-1 (GLP-1), secreted by intestinal L-cells, also plays a crucial role in regulating glucose homeostasis. Within the family pedigree reported by our research team, three diabetic patients can be categorized as the GLP-1 secretion-deficient subtype according to the refined classification of type 2 diabetes mellitus proposed by Zou Dajin. Both calcium ions and amino acids, particularly aromatic amino acids, function as strong agonists of the CaSR. CaSRs localized on the intestinal epithelium are capable of modulating GLP-1 secretion by sensing calcium ions and amino acids within the intestinal lumen. The marked reduction up to 70% in GLP-1 secretion induced by CaSR antagonists, such as Calhex 231 and NPS2143, and the substantial increase in GLP-1 secretion mediated by the CaSR agonist calindol, collectively underscore the indispensable role of intestinal CaSR in maintaining blood glucose homeostasis.^5^ The alteration of the spatial structure of the CaSR weakens the secretion of GLP-1 mediated by calcium ions and amino acids, which may partly account for the hyperglycemia observed in the three individuals with *CASR* mutations within this pedigree. However, since we have not carried out further investigations into insulin, GLP-1, and glucose-dependent insulinotropic polypeptide secretion, we are unable to draw a definite conclusion regarding the relationship between this nonsense mutation and hyperglycemia.

In conclusion, we reported a novel nonsense mutation, c.1799 G > A, in the *CASR* gene within an FHH1 pedigree. Subsequent *in vitro* functional analyses revealed that this germline mutation led to a truncated form of CaSR and blunted the reaction of MAPK pathway. However, it remains ambiguous whether this genetic variant plays a role in the development of hyperglycemia.

## CRediT authorship contribution statement

**Yang Tian:** Writing – review & editing, Writing – original draft, Project administration, Methodology, Investigation, Funding acquisition, Formal analysis, Data curation, Conceptualization. **Bingyang Liu:** Writing – original draft, Methodology, Data curation, Conceptualization. **Yang Xudan:** Methodology. **Ruojun Qiu:** Data curation. **Zhang Shaojun:** Writing – original draft, Conceptualization. **Huang Haoshu:** Methodology, Conceptualization. **Wu Fang:** Writing – review & editing, Methodology, Investigation, Data curation, Conceptualization. **Fenping Zheng:** Writing – review & editing, Writing – original draft, Data curation, Conceptualization.

## Ethics declaration

The study was approved by the Ethics Committee of the Fourth Affiliated Hospital, Zhejiang University School of Medicine (K2021125). All participants provided written informed consent after a detailed explanation of the study protocol.

## Funding

This work was supported by the 10.13039/501100014759Medical Scientific Research Foundation of Zhejiang Province, China (No. 2022KY197).

## Conflict of interests

The authors declared no conflicting interests.

## Appendix A Supplementary data

Multimedia component 1 Multimedia component 1

Figure S1 Construction and confirmation of mutant *CASR* plasmid and its expression. **(A)** Vector map of *CASR* W600∗ plasmid tagged with an N-terminal FLAG epitope. The human *CASR* expressing plasmid was generated by inserting a PCR-amplified human *CASR* cDNA (NM_001178065.1) fragment into vector GV741 (CMV enhancer-MCS-polyA-EF1A-mCherry-sv40-puromycin, Shanghai Genechem Co., Ltd., China) using KpnI and AgeI restriction enzymes, and a premature stop codon (TAG) was introduced at position c.1799 via overlap PCR to get *CASR* W600∗ plasmid. Primes are listed in the supplementary materials. Sanger sequencing confirmed the correct insertion of both the FLAG tag and the nonsense mutation. **(B)** Validation of mutant CaSR expression. To confirm the successful expression of the mutant *CASR* in transfected cells, total RNA was extracted 48 h post-transfection, reverse transcription was performed, and then it was subjected to PCR amplification. As expected, Sanger sequencing of MW and MUT revealed overlapping peaks of A and G at position c.1799, while the WILD exhibited a single peak of G. The amplifying primer pair is listed in the supplementary materials. CASR, calcium sensing receptor; MW, HEK293 cells transfected with 1 μg mutant *CASR* plasmid together with 1 μg wild *CASR* plasmid; MUT, HEK293 cells transfected with 2 μg mutant *CASR* plasmid; WILD, HEK293 cells transfected with 2 μg wild *CASR* plasmid; CON, HEK293 cells transfected with 2 μg of control plasmid. Figure S1
