# Supplementary material for: Identification and functional characterization of a novel nonsense mutation of CASR gene in a familial hypocalciuric hypercalcemia pedigree
Source: Genes Dis. 2025 Jul 25;13(3):101781. doi: 10.1016/j.gendis.2025.101781 (PMC12856587; doi:10.1016/j.gendis.2025.101781)
Supplement: Multimedia component 1 [file mmc1.docx]

**Materials and Methods**

**Subjects**

The study was approved by Ethics Committee of the Fourth Affiliated Hospital, Zhejiang University School of Medicine. All participants provided written informed consent after a detailed explanation of the study protocol.

**Molecular Screening of FHH**

Genomic DNA was extracted from peripheral blood leukocytes, and gene sequencing was performed by Dian Diagnostics Group CO., LTD. The proband underwent whole exons sequencing, and her family members received the mutation site of *CASR* gene verification.

**Plasmid Construction and Site-directed Mutagenesis**

The 3flag-tagged human *CASR* expressing plasmid (pcDNA/h*CASR*) was generated by inserting a PCR-amplified human *CASR* cDNA fragment into vector GV741 (CMV enhancer-MCS-polyA-EF1A-mCherry-sv40-puromycin, Shanghai Genechem Co., Ltd) using KpnI and AgeI restriction enzyme. Amplifying primers:

F: 5’-GTGGATCCGAGCTCGGTACCCGCCACCATGGCATTTTATAGCTGCTGCTGGGTCC-3’

R: 5’-AAAGATATTTTATTACCGGTTTATGAATTCACTACGTTTTCTGTAAC-3’.

The pcDNA/h*CASR* was used as a template to construct single nucleotide substitution mutant, *CASR* 1799G>A, through overlap PCR with the following 4 primes:

P1: 5’- ACCACACTGGACTAGTGGATCCGAGCTCGGTACCCGCCACCATGGCATTTTATAGCTG-3’

P2: 5’- GCAGGAGGTGTGGTTCTCATTGGACTAGAAGTCATCTGGGCACTTG-3’

P3: 5’- CAAGTGCCCAGATGACTTCTAGTCCAATGAGAACCACACCTCCTGC-3’

P4: 5’- CACACAGATGTAATGAAAATAAAGATATTTTATTACCGGTTTATGAATTCACTACG-3’.

Fragment A and B were amplified using primer pairs P1/P2 and P3/P4, respectively, from the 3flag-tagged human *CASR*. Subsequently, fragment C was amplified with primers P1/P4, employing fragments A and B as templates. Fragment V, digested from plasmid GV741 with KpnI/AgeI enzymes, was then recombined with fragment C to generate the 3flag-*CASR* W600* plasmid (*CASR* W600*). The sequence diagram of this constructed plasmid is presented in Figure S1 (A).

**Cell Culture, Plasmid Transfection**

HEK-293 cells were cultured in DMEM with 10% fetal bovine serum (FBS) and transfected with plasmid DNA using PolyJet^TM^ according to the manufacturer’s protocol. Cells planked in 6-well plate were transfected with 1ug mutant *CASR* plasmid together with 1ug wild *CASR* plasmid (MW) to mimic heterozygous mutation of *CASR*, 2ug mutant *CASR* plasmid (MUT), 2ug wild *CASR* plasmid (WILD) and 2ug of control plasmid (CON), cells were visualized with fluorescence microscope to check transfection efficiency. Proteins and mRNA were extracted 48 hours after transfection, mRNA then underwent reverse transcription and amplification with prime pair: F: 5’-TGCTGCTTTGAGTGTGTGGA-3’, R: 5’-GCACAAAGGCTGTCAGGAAA-3’ for nucleotide sequence identification to confirmed successfully plasmid DNA transfection, the sequence results were in Figure S1 (B).

**Analysis of CASR Protein Expression**

Cell membrane expression of CaSR was assessed through immunofluorescence and Western blot. The HEK293 cells were transfected with plasmid, then incubated with anti-Flag antibody overnight after fixation with paraformaldehyde, DAPI counterstaining and observed under confocal microscopy. The protein expression of *CASR* and Flag was assessed through Western blot by anti-CaSR (ThermoFisher: MA1-934, corresponding to amino acids 215-235 of human *CASR*) and anti-Flag antibody, hydrophobic and hydrophilic protein were also extracted and enriched by CelLytic ™ MEM Protein Extraction Kit (Sigma-Aldrich: CE0050) for analysis of protein membrane expression.

**Assay of CaSR Signal Transduction**

The ability of mutant type CaSR to activate the MAPK pathway was evaluated by examining the phosphorylation of ERK1/2. HEK293 cells transfected with wild and mut-type CASR cDNA plasmids were hungry from FBS for 6 hours and treated with increasing concentration of eCa^2+^ (0, 1, 2, 3, 4, 6, 8mM) for 15 minutes, proteins were harvested for immunoblotting to analyze the phosphorylation of ERK1/2. The ratio of pERK1/2 over total ERK1/2, expressed as a percentage of the maximum response value for each concentration measured on the basis of bands intensity, on the y-axis, and concentrations of eCa^2+^ were plotted on x-axis, subsequently, the 2 curves were compared (values are plotted as the mean ± SEM, P-values indicate the unpaired MW t-test on WILD for each concentration). The data presented are the cumulative results of n = 4 independent biological replicates.

Table S1. Characteristics of the pedigree

|  | II:2 | II:3 | II:5 | II:7 | III:1 | III:2 |
| --- | --- | --- | --- | --- | --- | --- |
| Gender | F | F | M | M | M | F |
| Age, y | 59 | 53 | 61 | 69 | 21 | 29 |
| Height, cm | 157 | 153 | 169 | 164 | 172 | 160 |
| Weight, Kg | 56 | 61 | 73 | 69 | 76 | 63 |
| S-Ca, mM  (2.11-2.52) | 2.96 | 2.24 | 2.87 | 2.79 | 2.72 | 2.39 |
| S-PO4, mM  (0.85-1.51) | 1.11 | 1.21 | 1.25 | 0.93 | 0.99 | 1.08 |
| Ab, g/L (40.0-55.0) | 45.0 | 41.0 | 41.2 | 43.9 | 44.2 | 45.0 |
| 25(OH)D, ng/mL (≥20) | 25 | 16 | 22 | 24.5 | 23 | 19 |
| 24H U-Ca, mmol/d (2.50-7.50) | 2.20 | 6.85 | 1.23 | 0.74 | 2.04 | 7.80 |
| 24H U-Cr, umol/d (6300-13400) | 10918 | 9361 | 9647 | 10618 | 7915 | 10213 |
| CCCR | 0.0051 | 0.0214 | 0.0031 | 0.0022 | 0.0050 | 0.0207 |
| iPTH, pg/mL (15.0-65.0) | 22.5 | 28.8 | 26.2 | 28.4 | 31.7 | 35.4 |

Table S2. Mixed diet and C-peptide releasing test of hyperglycemia members of the pedigree. The fasting C-peptide (FPC) levels of the 3 subjects ranged from 0.5 to 0.67 nmol/L, and their peak secretion was approximately 3-4 times the basal level, indicating a reserved insulin secreting function.

|  | BG, mmol/L | | | | | C-peptide, nmol/L | | | | |
| --- | --- | --- | --- | --- | --- | --- | --- | --- | --- | --- |
| Time, min | 0 | 30 | 60 | 120 | 180 | 0 | 30 | 60 | 120 | 180 |
| II:2 | 7.16 | 13.09 | 19.02 | 23.27 | 28.52 | 0.50 | 0.82 | 1.22 | 1.61 | 1.83 |
| II:5 | 8.66 | 13.40 | 20.32 | 25.16 | 22.38 | 0.52 | 0.68 | 0.96 | 1.40 | 1.38 |
| II:7 | 7.48 | - | - | 15.43 | - | 0.67 | - | - | 1.97 | - |

Table S3. Nonsense mutation in *CASR* reported in FHH1. Average of S-Ca of mutations located in exon 3-4 which will undergo NMD is 2.625±0.024mM (presented as mean ± SEM), lower than mutations happened in exon 7 (2.827±0.047mM), concentrations of S-Ca in 2 groups were compared in t-test, *p*=0.0042.

| Codon | Amino acid substitution | Exon | NMD or not | Location of mutation | S-Ca, mM | CCCR |
| --- | --- | --- | --- | --- | --- | --- |
| 25 | R25*^1^ | 2 | NMD | ECD | 2.68 | 0.0006 |
| 101 | C101*^2^ | 3 | NMD | ECD | 2.55 | 0.171g/24h |
| 227 | A227*^2^ | 4 | NMD | ECD | 2.57 | 0.049-0.057g/24h |
| 293 | W293*^2^ | 4 | NMD | ECD | 2.62 | - |
| 323 | K323*^1^ | 4 | NMD | ECD | 2.62 | 0.0068 |
| 352 | W352*^3^ | 4 | NMD | ECD | 2.7 | - |
| 573 | Y573*^3^ | 7 | Non-NMD | ECD | moderate | - |
| 583 | N583*^4^ | 7 | Non-NMD | ECD | modest | low |
| 600 | W600* | 7 | Non-NMD | ECD | 2.72-2.96 | 0.0051 |
| 607 | S607*^5^ | 7 | Non-NMD | ECD | 2.79 | 0.008 |
| 612 | F612*^6^ | 7 | Non-NMD | ECD | - | - |
| 648 | R648*^7^ | 7 | Non-NMD | TMD | 2.83 | 0.0045-0.0054 |
| 718 | W718*^8^ | 7 | Non-NMD | TMD | 2.88 | 0.006 |
| 892 | K892*^2^ | 7 | Non-NMD | ICD | 2.67 | - |

1. Ward BK, Magno AL, Blitvich BJ, et al. Novel mutations in the calcium-sensing receptor gene associated with biochemical and functional differences in familial hypocalciuric hypercalcaemia. *Clin Endocrinol*. 2006;64(5):580-587. doi:10.1111/j.1365-2265.2006.02512.x

2. Dershem R, Gorvin CM, Metpally RPR, et al. Familial Hypocalciuric Hypercalcemia Type 1 and Autosomal-Dominant Hypocalcemia Type 1: Prevalence in a Large Healthcare Population. *The American Journal of Human Genetics*. 2020;106(6):734-747. doi:10.1016/j.ajhg.2020.04.006

3. Nissen PH, Christensen SE, Heickendorff L, Brixen K, Mosekilde L. Molecular Genetic Analysis of the Calcium Sensing Receptor Gene in Patients Clinically Suspected to Have Familial Hypocalciuric Hypercalcemia: Phenotypic Variation and Mutation Spectrum in a Danish Population. *The Journal of Clinical Endocrinology & Metabolism*. 2007;92(11):4373-4379. doi:10.1210/jc.2007-0322

4. Pidasheva S, Grant M, Canaff L, Ercan O, Kumar U, Hendy GN. Calcium-sensing receptor dimerizes in the endoplasmic reticulum: biochemical and biophysical characterization of CASR mutants retained intracellularly. *Human Molecular Genetics*. 2006;15(14):2200-2209. doi:10.1093/hmg/ddl145

5. Pearce SH, Trump D, Wooding C, et al. Calcium-sensing receptor mutations in familial benign hypercalcemia and neonatal hyperparathyroidism. *J Clin Invest*. 1995;96(6):2683-2692. doi:10.1172/JCI118335

6. Hannan FM, Nesbit MA, Zhang C, et al. Identification of 70 calcium-sensing receptor mutations in hyper- and hypo-calcaemic patients: evidence for clustering of extracellular domain mutations at calcium-binding sites. *Human Molecular Genetics*. 2012;21(12):2768-2778. doi:10.1093/hmg/dds105

7. Jap TS, Wu YC, Jenq SF, Won GS. A Novel Mutation in the Calcium-Sensing Receptor Gene in a Chinese Subject with Persistent Hypercalcemia and Hypocalciuria1. *The Journal of Clinical Endocrinology & Metabolism*. 2001;86(1):13-15. doi:10.1210/jcem.86.1.7149

8. Rus R, Haag C, Bumke-Vogt C, et al. Novel Inactivating Mutations of the Calcium-Sensing Receptor: The Calcimimetic NPS R-568 Improves Signal Transduction of Mutant Receptors. *The Journal of Clinical Endocrinology & Metabolism*. 2008;93(12):4797-4803. doi:10.1210/jc.2008-1076
