# Supplementary figures and images for: Identification and functional characterization of a novel nonsense mutation of CASR gene in a familial hypocalciuric hypercalcemia pedigree
Source: Genes Dis. 2025 Jul 25;13(3):101781. doi: 10.1016/j.gendis.2025.101781 (PMC12856587; doi:10.1016/j.gendis.2025.101781)

A

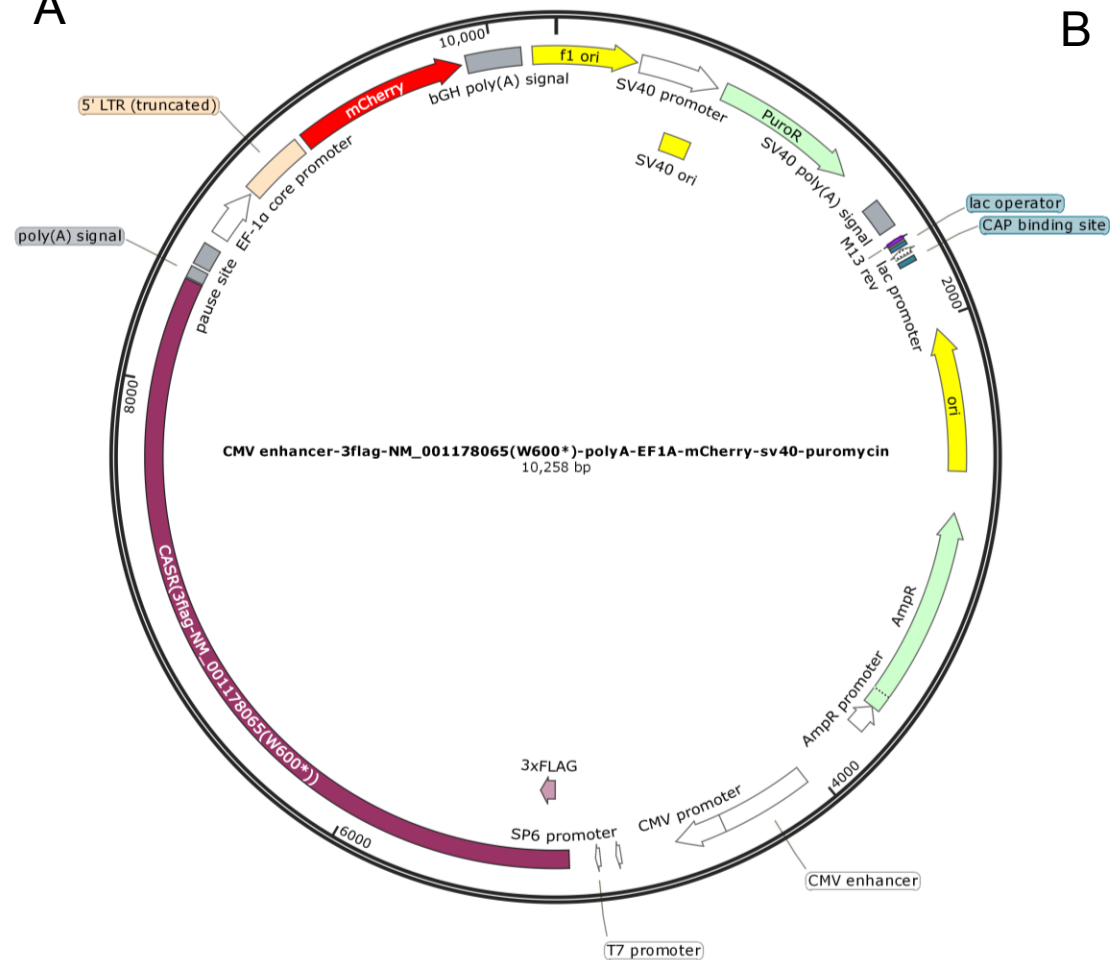

B

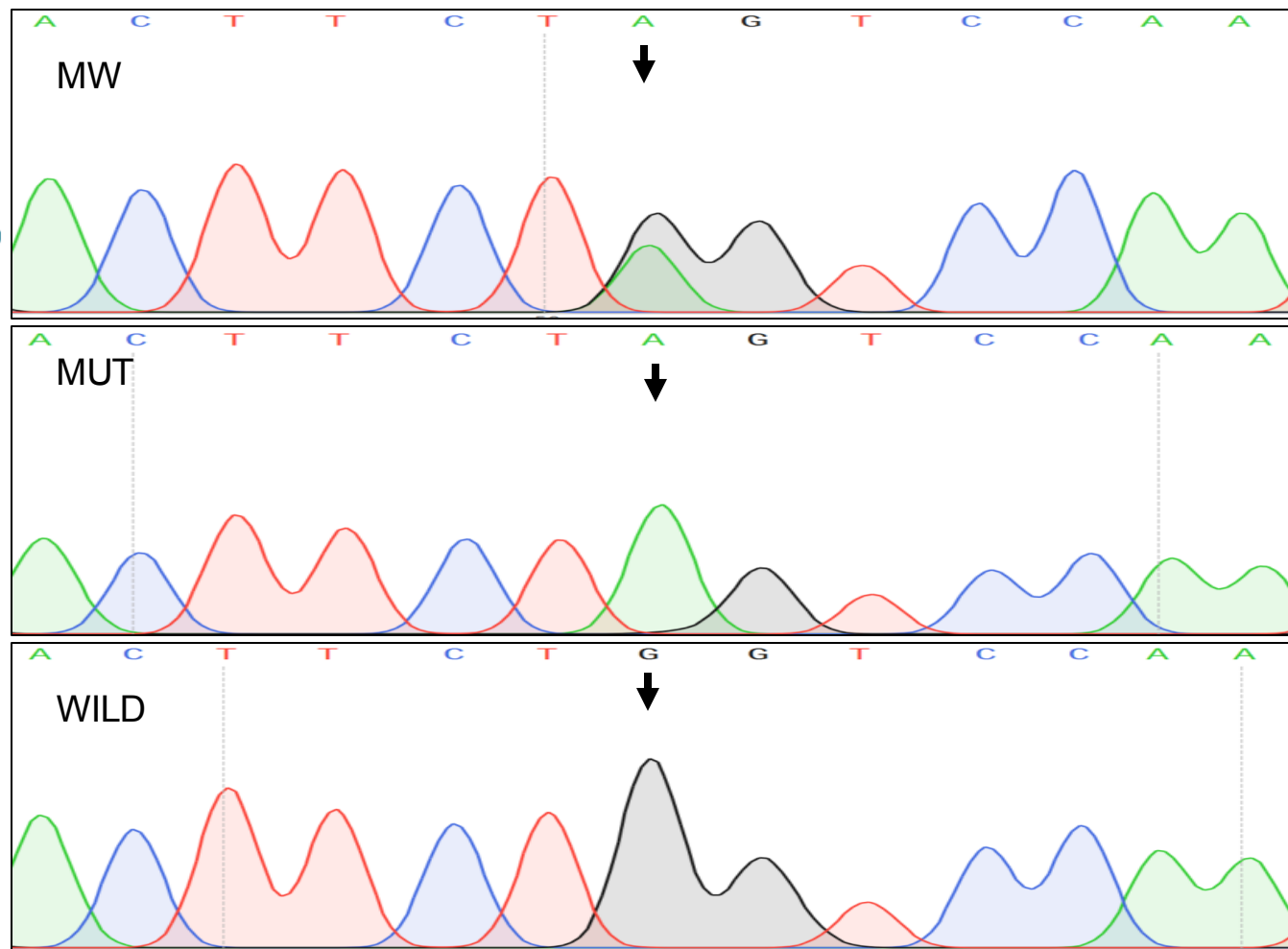

Supplement: Figure S1 — Construction and confirmation of mutant CASR plasmid and its expression. (A) Vector map of CASR W600∗ plasmid tagged with an N-terminal FLAG epitope. The human CASR expressing plasmid was generated by inserting a PCR-amplified human CASR cDNA (NM_001178065.1) fragment into vector GV741 (CMV enhancer-MCS-polyA-EF1A-mCherry-sv40-puromycin, Shanghai Genechem Co., Ltd., China) using KpnI and AgeI restriction enzymes, and a premature stop codon (TAG) was introduced at position c.1799 via overlap PCR to get CASR W600∗ plasmid. Primes are listed in the supplementary materials. Sanger sequencing confirmed the correct insertion of both the FLAG tag and the nonsense mutation. (B) Validation of mutant CaSR expression. To confirm the successful expression of the mutant CASR in transfected cells, total RNA was extracted 48 h post-transfection, reverse transcription was performed, and then it was subjected to PCR amplification. As expected, Sanger sequencing of MW and MUT revealed overlapping peaks of A and G at position c.1799, while the WILD exhibited a single peak of G. The amplifying primer pair is listed in the supplementary materials. CASR, calcium sensing receptor; MW, HEK293 cells transfected with 1 μg mutant CASR plasmid together with 1 μg wild CASR plasmid; MUT, HEK293 cells transfected with 2 μg mutant CASR plasmid; WILD, HEK293 cells transfected with 2 μg wild CASR plasmid; CON, HEK293 cells transfected with 2 μg of control plasmid. [file mmc2.pdf]
